# The psychological impact of pediatric movement disorders on families: brief report

**DOI:** 10.3389/fped.2026.1754852

**Published:** 2026-06-05

**Authors:** Hanin AlGethami

**Affiliations:** Pediatric Neurology Department, National Neuroscience Institute, King Fahad Medical City, Riyadh, Saudi Arabia

**Keywords:** caregiver burden, family dynamics, family strain, Middle East, multidisciplinary care, pediatric neurology, social psychology, stigma

## Abstract

**Background:**

Pediatric movement disorders impose substantial physical, emotional, and psychological stress on affected children and their families. Despite growing recognition of caregiver strain in chronic pediatric conditions, the unique burdens of movement disorders remain underexplored. To our knowledge, no prior study has comprehensively examined these impacts psychologically on families.

**Objective:**

To evaluate the psychological impact of pediatric movement disorders on caregivers and families and highlight evidence-based coping strategies and systemic support mechanisms.

**Methods:**

A narrative review was conducted using PubMed and Scopus databases up to January 2026, focusing on studies addressing caregiver burden, family impact, and coping in pediatric movement disorders and similar chronic conditions.

**Results:**

Caregivers commonly experience chronic stress, anxiety, depression, and physical health strain. Supportive factors—such as peer networks, multidisciplinary care, psychological counseling, and community integration—help mitigate these negative effects. Findings also highlight the influence of sociocultural and religious contexts in the Gulf region on caregiving experiences and coping patterns, underscoring the need for tailored, family-centered assessment approaches. A family-centered questionnaire designed to capture movement type–specific challenges and caregiver experiences is currently under development, with formal psychometric validation planned in future studies.

## Introduction

Paediatric movement disorders encompass a diverse spectrum of neurological conditions characterized by disruptions in voluntary and involuntary movement, including dystonia, chorea, tics, myoclonus, and parkinsonism. Although these disorders primarily affect the child, they impose substantial psychosocial burdens on the entire family unit. Caregivers of children with chronic neurological conditions—such as epilepsy, cerebral palsy (CP), and neurodevelopmental disorders—have consistently reported elevated levels of stress, anxiety, depression, and diminished quality of life (1–3).

The complexity of movement disorders—marked by diagnostic uncertainty, symptom fluctuation, stigma, and prolonged caregiving—demands focused attention. Importantly, the psychological burden experienced by families is not uniform. It varies considerably depending on factors such as the duration, clinical complexity, and type of the movement disorder, as well as the underlying etiology. Progressive or treatment-resistant conditions may impose greater long-term stress and uncertainty, whereas milder or self-limiting disorders may exert a comparatively lower burden (1). Addressing these varying caregiver experiences requires flexible and individualized support approaches. Recognizing this heterogeneity allows clinicians, mental health professionals, and support networks to better align interventions with specific family needs. To our knowledge, no previous study has comprehensively examined the psychological impact of paediatric movement disorders on families, including coping strategies and systemic support mechanisms.

This brief narrative report aimed to examine the psychological impact of paediatric movement disorders on caregivers and siblings, explore disruptions in family and social dynamics, and suggest evidence-based coping strategies aimed at supporting affected families.

## Methodology

A narrative review was conducted using literature searches in PubMed and Scopus from database inception to January 2026. Search terms included combinations of “paediatric movement disorders,” “caregiver burden,” “psychological impact,” “family stress,” and “coping.” Given the limited direct literature on pediatric movement disorders, evidence from epilepsy, cerebral palsy, and neurodevelopmental disorders was also incorporated when psychosocial mechanisms were comparable. Although not movement disorders *per se*, these conditions share chronicity, neurologic disability, and caregiving demands that parallel many aspects of the movement disorder experience.

Inclusion criteria comprised English-language studies involving caregivers or families of children with movement disorders or comparable neurological conditions. Exclusion criteria included studies focused solely on adults, single-case reports, and articles lacking a family or psychological component. Approximately 100 articles were screened, of which 39 were directly relevant to paediatric movement disorders or closely related conditions.

Data were extracted narratively and synthesized thematically across domains such as emotional well-being, family dynamics, social stigma, and coping strategies. Existing validated instruments—such as the Parenting Stress Index (PSI), Zarit Burden Interview (ZBI), and Caregiver Strain Questionnaire (CSQ) (4–6)—were reviewed for conceptual comparison. However, these tools are not specifically designed to capture the movement type–specific psychological and social dimensions relevant to pediatric movement disorders, including dystonia, tics, and chorea.

To address this gap, we propose a family-centered, domain-based questionnaire tailored to pediatric movement disorders. At present, this tool is conceptual and has not undergone formal psychometric validation. Future research will focus on systematic validation, including assessment of reliability, construct validity, and cross-cultural applicability, as part of a planned second-phase study.

## Psychosocial impact on families

### Caregiver emotional well-being

Caregivers frequently endure chronic psychological stress, characterized by persistent anxiety, depression, and emotional exhaustion. The unpredictable nature of movement symptoms and concerns regarding long-term prognosis contribute to heightened emotional reactivity (7). Studies in epilepsy and CP reveal analogous challenges, including hypervigilance and anticipatory grief (8, 9). Common emotional responses include helplessness and self-blame, often exacerbated by delays in diagnosis or limited treatment options (10). Extended caregiving duties may culminate in burnout and social withdrawal, aggravating mental health problems (11). Recent research further emphasizes that caregivers often face an elevated risk of depression and PTSD symptoms (12).

### Physical health and daily functioning

Beyond emotional distress, caregivers frequently experience deterioration in physical health, reporting fatigue, sleep disruption, musculoskeletal pain, and stress-related somatic symptoms, which ultimately affects their mental health (2). For instance, caregivers of children with CP demonstrate significantly poorer physical health outcomes relative to parents of typically developing children (9). This results in reduced overall quality of life for both parents and siblings. The cumulative burden of physical stressors compromises daily functioning and may predispose caregivers to chronic health conditions, thereby negatively impacting their psychological well-being (13).

## Family and social challenges

### Marital and partner relationships

Marital strain is prevalent among parents managing the needs of children with movement disorders. Divergent coping mechanisms may provoke conflict, with one parent assuming primary caregiving responsibilities while the other becomes emotionally or physically disengaged (7). Similar dynamics observed in families of children with autism or epilepsy show that uneven caregiving roles can foster emotional distance or separation (14, 15). The degree of marital stress often depends on the type and severity of the movement disorder, with progressive or complex conditions posing greater challenges (16).

### Siblings experience

Siblings often experience emotional neglect or are compelled to take on caregiving roles prematurely. Feelings of guilt, jealousy, and confusion are common, especially when their own needs are deprioritized (1). Without open dialogue, these children may internalize stress, leading to behavioral issues or academic difficulties (17, 18). Emerging studies highlight the need for sibling-focused psychosocial support to prevent adverse outcomes (19). These intrafamilial stressors often intersect with broader social challenges, amplifying family isolation and vulnerability.

### Social integration and stigma

Logistical barriers, coupled with societal misunderstanding of movement disorders, often limit families' social engagement, fostering isolation, shame, and frustration. Qualitative studies document stigma as a persistent barrier to community integration and a chronic stressor (20). Over time, social withdrawal erodes support networks, intensifying caregiver loneliness and vulnerability (21). Recent work emphasizes the efficacy of community-based stigma reduction programs in improving social inclusion (22). Moreover, families managing mild or self-limiting movement disorders may experience less disruption to social life than those caring for children with treatment-resistant or multisystem involvement.

### Economic and occupational impact

The economic impact of Paediatric movement disorders is considerable and highly variable, reflecting differences in symptom severity, treatment complexity, and access to supportive services. However, families face significant out-of-pocket costs related to medications, therapies, assistive devices, and travel to specialized centers (23). Caregiving demands frequently limit employment opportunities, leading to income loss and financial insecurity (24, 25). Limited availability of respite care, especially in resource-poor settings, exacerbates these challenges (26).

## Factors influencing the psychosocial impact on families

### Sociocultural and demographic context of caregiving

Sociocultural, demographic, and systemic factors play a pivotal role in shaping how families perceive, experience, and cope with pediatric movement disorders. In the Middle East and Gulf region, reliance on faith and belief in divine will often serves as an important coping mechanism, fostering resilience and acceptance. At the same time, families may continue to experience emotional distress related to uncertainty about diagnosis and long-term outcomes, particularly in chronic or complex conditions.

Cultural norms, including strong extended family structures, can provide essential emotional and practical support, yet may simultaneously contribute to pressure, misconceptions, or encouragement toward non-medical interventions. Community-related stigma surrounding neurological disorders further compounds caregiver stress and may limit social engagement (27, 28).

Broader demographic and socioeconomic variables also influence caregiver burden. Families in low- and middle-income countries (LMICs) frequently experience greater strain due to limited access to specialized services, financial constraints, and reduced availability of rehabilitation resources compared to those in high-income settings. Educational level is another key determinant, as higher health literacy enables caregivers to better navigate healthcare systems and advocate for their children. Additionally, caregiver characteristics such as age and sex impact coping capacity, with mothers more commonly assuming primary caregiving roles and reporting higher levels of psychological distress (29).

### Clinical and phenomenological variability of movement disorders

The nature and classification of pediatric movement disorders play a pivotal role in shaping the degree and character of caregiver burden. Organic movement disorders, including dystonia, cerebral palsy, and neurodegenerative conditions, are typically associated with chronic physical disability, complex medical needs, and long-term care requirements. These features often impose a sustained and cumulative strain on caregivers, encompassing both physical and emotional dimensions. In contrast, functional movement disorders (30), despite the absence of identifiable structural pathology—are frequently characterized by diagnostic ambiguity and fluctuating symptom patterns, which can generate significant uncertainty, frustration, and psychological distress among caregivers.

Moreover, the phenomenological diversity of movement disorders further contributes to variability in caregiver experience. Different manifestations, such as tremor, stereotypies, and tic disorders, differ in their functional impact and social visibility. Symptoms that are highly conspicuous or poorly understood by the public may expose families to increased scrutiny, stigma, and social discomfort. As a result, caregiver burden is not solely determined by disease severity, but also by the visibility, interpretability, and societal perception of the child's symptoms, highlighting the complex interplay between clinical characteristics and psychosocial context (16, 31, 32, 33).

Pediatric movement disorders impose a multifactorial psychosocial burden that extends beyond the child to affect the entire family. Caregiver stress is shaped by disease characteristics as well as socio-demographic, cultural, and contextual factors, with variability across different movement disorder types influencing experiences such as uncertainty and stigma. These findings highlight the need for a structured, family-centered approach. Given the substantial and heterogeneous impact on families, tailored, evidence-based support strategies are essential to enhance resilience and overall well-being.

## Coping strategies and support systems

### Multidisciplinary clinical and counselling interventions

A multidisciplinary approach involving neurologists, psychologists, physiotherapists, and social workers is critical to addressing both clinical and psychosocial needs (23). Movement disorders are complex, often requiring coordinated care that not only targets motor symptoms but also supports the psychological well-being of families. Psychological counselling equips caregivers with tools for emotional regulation, stress management, and resilience building, helping them cope with uncertainty, fluctuating symptoms, and long-term caregiving demands. Family counselling further promotes open communication, shared problem-solving, and reduced intra-family conflict, ultimately enhancing cohesion (34). Educational initiatives complement these therapeutic strategies by increasing disorder literacy, clarifying treatment expectations, and improving communication with healthcare professionals. Strengthening caregiver knowledge reduces helplessness and fosters more active engagement in treatment planning. Importantly, recent evidence highlights the value of telehealth-based counselling, which increases accessibility for families in remote or underserved regions while also reducing financial and logistical barriers to care (35, 36).

### Peer support network

Peer support networks—whether virtual or in-person—provide families with emotional validation, social connection, and opportunities to exchange practical caregiving strategies (34). These networks help normalize families' experiences and reduce feelings of isolation by connecting individuals facing similar challenges. Recent advancements in digital technology, including moderated online forums, webinars, and mobile applications, have further expanded access to high-quality peer support and shared experiential knowledge (36). Such platforms offer flexible, stigma-free environments where caregivers can safely discuss concerns, seek advice, and build supportive communities that reinforce resilience.

### Social inclusion and community integration

Beyond the family, schools and community organizations play a vital role in promoting social inclusion and reducing stigma. Inclusive education policies, disability awareness campaigns, and adaptive extracurricular activities not only encourage the child's participation but also reduce societal misconceptions about movement disorders. These inclusive environments provide psychosocial benefits to the entire family, alleviating parental anxiety while also promoting sibling involvement and acceptance. New research underscores the importance of tailored school-based interventions for children with movement disorders, which can enhance social participation, improve academic outcomes, and support mental health in both the child and family unit (22, 37).

### Respite care and self-care strategies

Structured respite care provides caregivers with essential time for rest, medical appointments, or personal pursuits, which is crucial for sustaining long-term caregiving (3). Without such breaks, families are at risk of burnout, which exacerbates both physical and psychological distress. Encouraging parents to engage in hobbies, physical activity, and social interaction helps preserve their identity outside of the caregiving role and strengthens emotional resilience. Additionally, routine family meetings or check-ins can serve as safe spaces for emotional expression, shared problem-solving, and reaffirming mutual support within the household (38). This combination of formal respite services and intentional self-care strategies ensures more sustainable caregiving while promoting overall family well-being.

### Policy-level supports and systemic interventions

In addition to family- and community-based strategies, broader policy initiatives are vital to sustain long-term caregiving. Expanding insurance coverage to include rehabilitation therapies, psychological counselling, and respite services can reduce the financial burden on families. Government-subsidized respite programs and inclusive education mandates further ensure equitable access to care and learning opportunities for children with movement disorders. Disability awareness campaigns and national registries also play key roles in reducing stigma, guiding resource allocation, and informing the design of targeted psychosocial interventions. Embedding these supports within health and education systems complements family-centered approaches, creating a more sustainable framework for resilience and well-being (39).

### Limitations

This narrative review may not have captured all relevant studies, and no formal quality appraisal or risk-of-bias assessment was conducted. Some findings were extrapolated from literature on other chronic pediatric neurological conditions (e.g., epilepsy, cerebral palsy), which may limit their specificity to pediatric movement disorders. Considerable heterogeneity across study populations, disorder types, and outcome measures further restricts direct comparisons and prevents quantitative synthesis. Limiting the search to English-language articles indexed in PubMed and Scopus may have excluded regionally published or non-indexed research. Additionally, grey literature and academic theses were not included, despite their potential relevance in low- and middle-income or non-English-speaking contexts. Despite these limitations, the consistency of themes across diverse sources underscores the need for standardized, family-centered assessment frameworks tailored to pediatric movement disorders.

### Clinical implications

The findings of this review highlight the importance of incorporating family-centered practices into the routine care of children with pediatric movement disorders. Routine screening of caregiver mental health, family dynamics—including the experiences of siblings—and the effects of social stigma should become standard components of clinical encounters. Early referral to psychology or social work teams is essential to ensure timely intervention and support. Embedding family-focused questions into routine clinic visits, and fostering collaboration with schools, community organizations, and allied health services, may help mitigate long-term psychosocial morbidity. Identifying high-risk families proactively enables clinicians to provide tailored support, reduce caregiver distress, and promote healthier family functioning in the context of pediatric movement disorders.

## Conclusion

Pediatric movement disorders are associated with a substantial and multifaceted psychological burden that extends beyond the affected child to impact the entire family unit. Caregivers and siblings frequently experience complex emotional, social, and practical challenges, underscoring the need for coordinated, multidisciplinary support. Addressing these needs through integrated medical, psychological, educational, and community-based approaches is essential to enhance family well-being and long-term outcomes.

Importantly, the caregiving experience is not uniform and varies according to disease duration, clinical complexity, underlying etiology, and prognosis. These factors should be carefully considered when developing individualized and context-sensitive support strategies.

To enable more standardized assessment of caregiver burden, a family-centered questionnaire tailored to pediatric movement disorders is currently under development. This tool is designed to provide a structured and scalable framework for evaluating caregiver experiences and guiding targeted, family-centered interventions. At present, the questionnaire remains in the conceptual phase, and future research will focus on rigorous psychometric validation, including evaluation of reliability, construct validity, and cross-cultural applicability.

Overall, this brief report highlights the urgent need for culturally sensitive, disorder-specific frameworks that not only address the clinical aspects of pediatric movement disorders but also prioritize family resilience and holistic, family-centered care.
